# Gelseminum Sempervirens in Facial Neuralgia

**Published:** 1877-09-20

**Authors:** 


GELSEMINUM SEMPERVIRENS IN FACIAL NEURALGIA. — Dr. Spencer Thomson sums up his experience with this drug, and finds it of most value in neuralgic affections of the superior and inferior maxillary nerves, especially the latter, and more particularly when the pain is referred to the teeth or alveoli. In almost every instance, speedy and thorough relief was given.

At the Nervous Infirmary, in this city, some trial has been given this drug, but not sufficiently to arrive at any perfectly safe conclusions. So far, however, it seems to be of more value in acute than chronic facial neuralgia. It has failed more than once. At the same time, Dr. Mitchell considers it rather a tricky drug. This same medicine was tried for chorea, but in the majority of cases the results were not favorable.

